# Association between the direct bilirubin to lymphocyte ratio and mortality in patients with necrotizing fasciitis: a retrospective cohort study

**DOI:** 10.3389/fmed.2026.1841323

**Published:** 2026-06-01

**Authors:** Jianghui Dong, Qianliu Li, Xiannian Shao, Shuliang Hua, Jialong Nong, Jili Lu, Keyi Ma, Liping Wang

**Affiliations:** 1 Department of Joint Surgery, Baise People's Hospital, Affiliated Southwest Hospital of Youjiang Medical University for Nationalities, Baise, Guangxi, China; 2 Department of Anaesthesiology, Baise People's Hospital, Affiliated Southwest Hospital of Youjiang Medical University for Nationalities, Baise, Guangxi, China; 3 Department of Hospital Development and Operations Management, Baise People's Hospital, Affiliated Southwest Hospital of Youjiang Medical University for Nationalities, Baise, Guangxi, China

**Keywords:** 28-day mortality, biomarker, Cox regression, direct bilirubin to lymphocyte ratio, necrotizing fasciitis, prognosis

## Abstract

**Background:**

Necrotizing fasciitis (NF) is a rapidly advancing and highly lethal infection of the soft tissues. We investigated the link between the direct bilirubin-to-lymphocyte ratio (DBLR) and 28-day mortality in NF cases.

**Methods:**

This single-center retrospective cohort study enrolled NF patients admitted to Baise People's Hospital between November 2019 and December 2025. To assess the prognostic value of DBLR, we employed restricted cubic spline (RCS) analysis, Kaplan–Meier analysis, multivariable Cox regression, subgroup analysis, and receiver operating characteristic (ROC) analysis.

**Results:**

A total of 184 patients were included. Patients in higher DBLR quartiles showed more severe hepatic, coagulation, and inflammatory abnormalities, together with higher 28-day mortality. After log_10_ transformation, RCS analysis showed a significant overall association between log_10_(DBLR) and 28-day mortality, with no evidence of non-linearity (*P* for overall < 0.001; *P* for non-linearity = 0.515). In Cox regression analysis, a higher log_10_(DBLR) was associated with increased 28-day mortality. This association remained significant after adjustment for age, shock, and LRINEC in Model 3 (HR, 2.119; 95% CI, 1.384–3.243; *P* < 0.001). Similar results were observed in the SOFA-adjusted and APACHE II-adjusted models. DBLR showed modest-to-acceptable discrimination for 28-day mortality, with an AUC of 0.733 (95% CI, 0.644–0.811).

**Conclusions:**

An enhanced DBLR is linked to an increased risk of mortality within 28 days in NF. It could serve as a simple additional biomarker for early risk evaluation.

## 1 Introduction

Necrotizing fasciitis (NF) is a serious infection of the soft tissue. It spreads quickly and can damage the tissue under the skin, including muscles and organs. Even though it happens rarely, with 0.72 to 9.2 cases for every 100,000 people each year, the associated mortality ranges from 20% to 76%, with 90-day case fatality rates nearing 18% to 25% even in well-equipped tertiary facilities (1–4). Notably, reported mortality ranges vary significantly across studies, with some cohorts reaching as high as 76% depending on clinical setting (1–4). The clinical characteristic is permanent tissue death caused by synergistic polymicrobial or monomicrobial invasion, when gas-forming organisms proliferate along low-resistance fascial planes within hours. The fast advancement indicates that any postponement in surgical débridement significantly deteriorates results. However, the nuanced, nonspecific initial appearance, sometimes indistinguishable from cellulitis or deep-space abscess, continues to be the primary obstacle to prompt identification and management (5–7). Recent epidemiological assessments indicate that NF remains a substantial national mortality burden, with septicemia being the most prevalent presenting complication (5). Prompt surgical intervention and broad-spectrum antibiotic treatment are fundamental to care, with other modalities, such as intravenous immunoglobulin, currently under investigation (8–12).

Initial scans, such as ultrasound, CT, and MRI can help diagnose quickly when things are unclear. Nevertheless, no imaging technique can replace surgical investigation and clinical judgment (8, 13). Significant work has focused on discovering prognostic biomarkers that might stratify mortality risk at presentation. The neutrophil-to-lymphocyte ratio (NLR) and platelet-to-lymphocyte ratio (PLR) have independent correlations with NF mortality in multivariate analysis, indicating systemic inflammatory-immune dysregulation associated with severe infection (14, 15). The systemic inflammation response index (SIRI) and C-reactive protein-to-albumin ratio (CAR) encompass complementary aspects of inflammation and nutritional–catabolic stress and have been shown to be independent predictors of NF incidence and severity (16). Indices derived from red cell distribution width, namely RAR, attained an AUC of 0.955 predicting in-hospital mortality in scrotal Fournier's gangrene (17), and the predictive value of RDW-derived measures has been further validated in sepsis and bacteremia cohorts (18–21). The LRINEC score was created to tell necrotizing fasciitis apart from other soft-tissue infections, has demonstrated inadequate sensitivity in several validation populations, and was not originally intended for mortality prediction (22, 23). Mortality predictors in necrotizing fasciitis (NF), including shock, increased creatinine levels, and hypoalbuminemia, have been identified in extensive institutional studies (24). Patients with diabetes mellitus represent a clinically important subgroup in NF (25). These parameters together reflect inflammatory activation or nutritional depletion, without directly addressing hepatic metabolic failure, a pathophysiological characteristic often seen in severe NF-related systemic impairment.

Serum direct bilirubin indicates the liver's ability to conjugate and eliminate bilirubin through the biliary system. In severe infections, its increase arises from many converging mechanisms: hepatocyte damage driven by inflammatory cytokines, cholestasis associated with sepsis, ischaemia-reperfusion injury due to circulatory compromise, and disseminated intravascular coagulation accompanied by microvascular blockage. Hypoalbuminemia is a marker of acute illness severity and has been associated with poor outcomes in critically ill patients (26). Lymphocytopenia is common in severe infection and sepsis and reflects apoptosis-mediated lymphocyte depletion and immune suppression (27). Beyond NF-specific cohorts, NLR and SIRI have also been studied in broader critical illness and sepsis populations (28, 29).

By integrating these two pathophysiologically distinct signals into a single composite ratio, DBLR simultaneously captures hepatobiliary dysfunction and immune cell depletion, thereby providing a more comprehensive index of systemic injury burden in NF than either constituent alone. To our knowledge, no prior research has explicitly investigated the correlation between DBLR and mortality in NF. Consequently, we performed a retrospective observational analysis encompassing 184 necrotizing fasciitis patients treated at a single institution over a ten-year period. The main purpose was to ascertain if admission DBLR is independently linked to 28-day all-cause mortality; the secondary objective was to examine the prognostic accuracy of DBLR against its individual components and the LRINEC score.

## 2 Methods

### 2.1 Data source

The Baise People's Hospital Ethics Review Committee approved this study (No. KY2026032036). Because the study was retrospective and used de-identified patient data, the committee decided not to seek informed permission. Every method adhered to the Declaration of Helsinki guiding principles.

### 2.2 Study design and population

**Figure 1:**
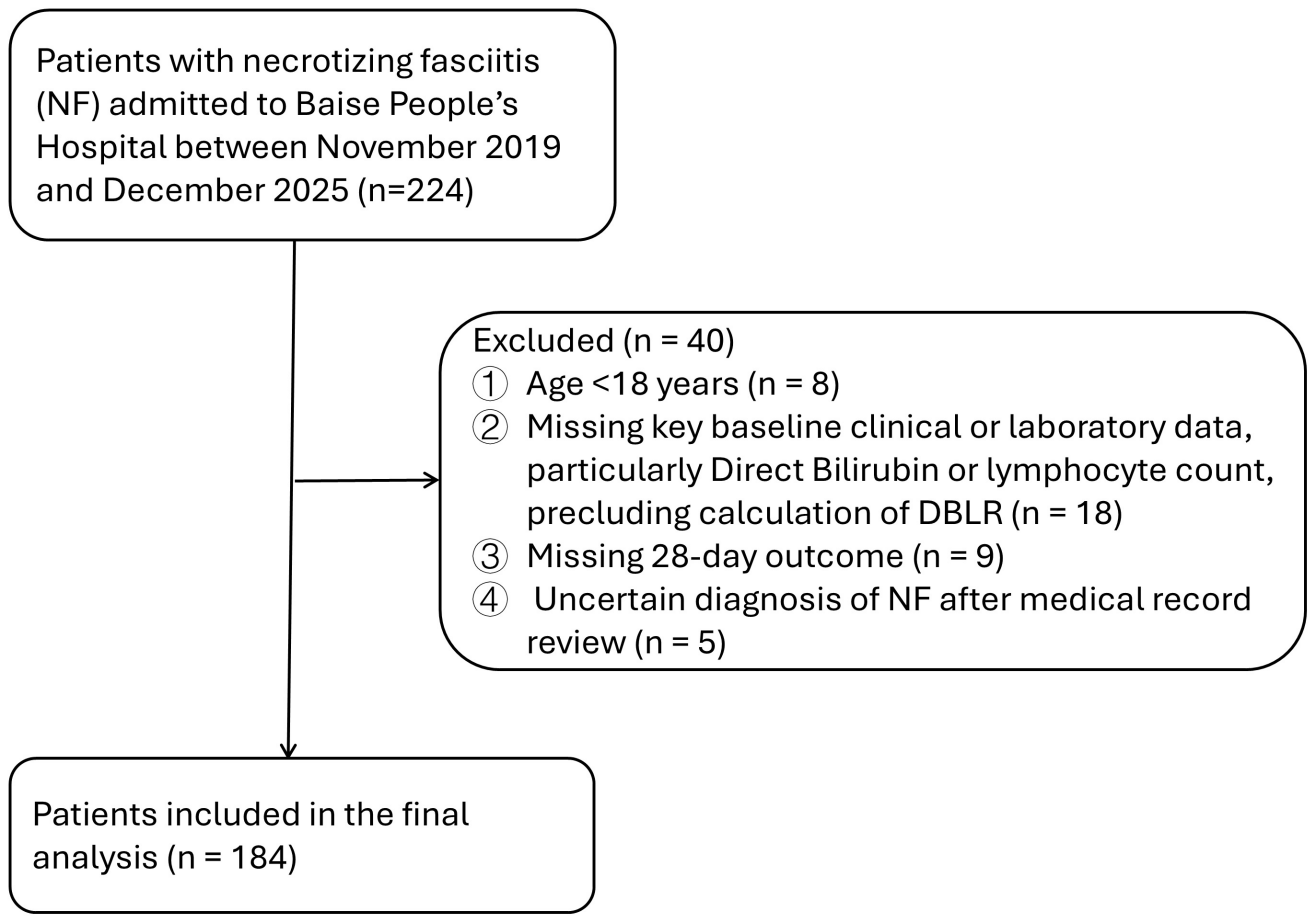
Flowchart of this study. A total of 224 patients diagnosed with NF were screened. Forty patients were excluded according to predefined criteria: age <18 years (*n* = 8), missing baseline clinical or laboratory data required for DBLR calculation (*n* = 18), unavailable 28-day outcome data (*n* = 9), and uncertain NF diagnosis after chart review (*n* = 5). The final analytic cohort included 184 patients. DBLR, direct bilirubin-to-lymphocyte ratio; NF, necrotizing fasciitis.

The STROBE standards were followed in the conduct of this retrospective observational study. Every patient who was admitted to Baise People's Hospital between November 2019 and December 2025 and had a verified diagnosis of NF underwent eligibility screening. (1) admission to Baise People's Hospital during the study period with a diagnosis of NF; (2) age ≥18 years; (3) NF confirmed based on clinical presentation and operative findings, supplemented by histopathological or microbiological evidence when available; (4) availability of complete baseline laboratory data, including direct bilirubin and absolute lymphocyte count sufficient to compute DBLR; and (5) availability of 28-day outcome data. Necrotizing fasciitis was diagnosed based on compatible clinical presentation, supportive imaging findings, and operative evidence. Characteristic intraoperative findings included fascial necrosis, gray necrotic tissue, dishwater-like fluid, loss of tissue resistance, absence of bleeding, and easy dissection along fascial planes. Histopathological and microbiological data were recorded when available but were not mandatory for inclusion. Patients were excluded when the totality of available clinical, radiological, operative, pathological, microbiological, and discharge documentation was insufficient to confirm the diagnosis. The following were the exclusion criteria: (1) age < 18 years; (2) direct bilirubin or lymphocyte count absent, which prevented DBLR calculation; (3) unavailable 28-day outcome data; and (4) unclear NF diagnosis after systematic record review. 184 individuals remained in the final analytic cohort after 40 of the 224 eligible patients who were first identified were eliminated for not meeting the eligibility requirements (8 were less than 18 years old; 18 had missing critical laboratory data; 9 lacked 28-day outcome data; and 5 had an uncertain diagnosis) (Figure 1).

### 2.3 Data collection

The laboratory and clinical data are from electronic medical records, including age, sex, comorbidities (hypertension, diabetes mellitus), clinical status at admission (shock, sepsis), complete blood count parameters (white blood cell count, neutrophil, lymphocyte, monocyte, and platelet counts, hemoglobin, hematocrit, red cell distribution width), liver function tests (albumin, AST, ALT, total bilirubin, direct bilirubin, indirect bilirubin, globulin, total protein, and uric acid), coagulation profile (activated partial thromboplastin time, fibrinogen, international normalized ratio, and prothrombin time), and inflammatory markers (C-reactive protein and procalcitonin). APACHE II, LRINEC, and SOFA severity ratings were calculated using admission data. All patients had blood samples taken within 24 h of admission. Missing data were assessed before model construction. Variables with more than 10% missing data were excluded from modeling. Consequently, serum creatine kinase (CK) was excluded from the multivariable analysis due to a missing rate of 45.1%. For variables with 10% or less missingness, multiple imputation by chained equations (MICE) was performed. The imputation model included baseline clinical variables, and laboratory variables. DBLR was calculated as serum direct bilirubin concentration (μmol/L) divided by absolute peripheral lymphocyte count ( × 10^9^/L), using the first available blood sample obtained within 24 h of admission. DBLR = Direct Bilirubin (μmol/L) / Lymphocyte Count ( × 10^9^/L).

### 2.4 Outcome definition

The primary endpoint was the 28-day all-cause mortality, calculated from the date of hospital admission. Outcomes were determined through in-hospital death records and structured follow-ups, conducted either via telephone or outpatient contact, on day 28.

### 2.5 Statistical analysis

Because raw DBLR exhibited a markedly right-skewed distribution, DBLR was transformed using the base-10 logarithm prior to Cox regression modeling, and the hazard ratio (HR) was reported per standard deviation increase in log10 (DBLR).

Continuous variables were shown as mean ± standard deviation or median (interquartile range), depending on the data. Categorical variables were presented as a number (percentage). Because raw DBLR exhibited a markedly right-skewed distribution, DBLR was transformed using the base-10 logarithm prior to Cox regression modeling, and the hazard ratio (HR) was reported per standard deviation increase in log10(DBLR). Quartile-based Cox models were retained only as secondary descriptive analyses because deaths were sparse in the lowest quartile. To examine the link between DBLR and 28-day mortality, RCS analysis was used. The log-rank test and Kaplan–Meier plots were used to see how long people lived in each group. DBLR was used as both a continuous variable and a category variable in Cox proportional hazards models. Specifically, Model 1 was adjusted for age; Model 2 was adjusted for age and shock; and Model 3 was adjusted for age, shock, and the Laboratory Risk Indicator for Necrotizing Fasciitis (LRINEC) score. To assess whether DBLR provided prognostic information beyond established severity scores, two additional severity-adjusted models were constructed. Model 4 was adjusted for SOFA, and Model 5 was adjusted for APACHE II. Subgroup analyses were performed stratified by age, sex, hypertension, diabetes, shock, and sepsis. Different studies were done for age, sex, high blood pressure, diabetes, shock, and sepsis. A ROC analysis was used to assess how well LRINEC, direct bilirubin, cell count, and DBLR could tell the difference between 28-day mortality. We used R version 4.5.2 to perform the statistical tests. A *P* value of less than 0.05 was considered statistically significant. Fisher's exact test was employed for categorical variables when expected cell counts were less than five.

## 3 Results

### 3.1 Patient characteristics

The final group had 184 patients. Out of these, 158 were men (85.9%). The mean age was 57.1 years, with a standard deviation of 11.9 years. The 28-day mortality rate was 21.7%, corresponding to 40 non-survivors. Non-survivors demonstrated significantly lower levels of total protein (*P* < 0.001, 53.0 vs. 60.2 g/L), albumin (*P* = 0.003, 25.6 vs. 29.1 g/L), lymphocyte count (1.1 vs. 1.6 × 10^9^/L, *P* < 0.001), and platelet count (177.5 vs. 286.2 × 10^9^/L, *P* < 0.001) compared to survivors. In non-survivors, direct bilirubin (*P* < 0.001, 10.1 vs. 4.3 μmol/L), urea (*P* = 0.007, 10.1 vs. 6.3 mmol/L), and AST (*P* = 0.001, 37.5 vs. 23.9 U/L) were much higher than in survivors. The incidence of sepsis (62.5% vs. 26.4%, *P* < 0.001) and shock (65.0% vs. 13.2%, *P* < 0.001) was significantly higher among non-survivors compared to survivors. The median DBLR was much higher in those who did not survive compared to those who did (8.0 [IQR 4.0–59.5] vs. 2.8 [IQR 1.1–6.6], *P* < 0.001). Among the 184 included patients, all had compatible clinical features and supportive imaging findings. Operative confirmation was available in 180 patients (97.8%). Histopathological confirmation was available in 92 patients (50.0%), and microbiological confirmation was available in 88 patients (47.8%). Overall, 180 patients (97.8%) had histopathological or microbiological confirmation. The anatomical distribution of NF was as follows: cervical NF in 4 patients (2.2%), extremity NF in 99 patients (53.8%), multiple-site involvement in 14 patients (7.6%), perineal/Fournier's gangrene in 38 patients (20.7%), and trunk/abdominal wall involvement in 29 patients (15.8%). Anatomical site was not significantly associated with 28-day mortality (*P* = 0.701) (Table 1). Therefore, it was not included in the primary multivariable Cox model.

**Table 1:** Baseline characteristics of patients with necrotizing fasciitis according to 28-day mortality status.

Variables	Total (*n* = 184)	Survivors (*n* = 144)	Non-survivors (*n* = 40)	*P*
Age (years), Mean ± SD	57.1 ± 11.9	56.5 ± 12.1	59.1 ± 11.3	0.227
Gender, *n* (%)				0.738
Female	26 (14.1)	21 (14.6)	5 (12.5)	
Male	158 (85.9)	123 (85.4)	35 (87.5)	
Urea (mmol/L), Median (IQR)	6.6 (4.6, 12.2)	6.3 (4.4, 9.7)	10.1 (5.9, 16.8)	0.007
Creatinine (μmol/L), Median (IQR)	82.0 (64.0, 129.0)	79.0 (64.0, 122.0)	88.5 (64.5, 160.0)	0.355
Uric acid (μmol/L), Mean ± SD	343.4 ± 162.9	332.6 ± 154.4	382.1 ± 187.4	0.089
Total protein (g/L), Mean ± SD	58.6 ± 10.2	60.2 ± 9.5	53.0 ± 11.0	< 0.001
Albumin (g/L), Mean ± SD	28.3 ± 6.7	29.1 ± 6.1	25.6 ± 8.3	0.003
Total bilirubin (μmol/L), Median (IQR)	11.9 (7.0, 20.9)	10.3 (6.4, 17.2)	16.6 (10.1, 30.6)	< 0.001
Direct bilirubin (μmol/L), Median (IQR)	5.0 (2.9, 9.9)	4.3 (2.6, 8.3)	10.1 (5.2, 21.9)	< 0.001
Indirect bilirubin (μmol/L), Median (IQR)	5.6 (3.4, 9.5)	5.3 (3.2, 9.2)	7.7 (4.7, 11.7)	0.007
Globulin (g/L), Mean ± SD	30.5 ± 8.0	31.6 ± 8.2	26.8 ± 6.1	< 0.001
Aspartate aminotransferase (U/L), Median (IQR)	25.0 (16.9, 45.2)	23.9 (15.0, 38.5)	37.5 (22.0, 68.2)	0.001
Alanine aminotransferase (U/L), Median (IQR)	26.0 (15.0, 47.0)	23.0 (14.0, 45.0)	35.3 (22.2, 49.8)	0.064
AST/ALT, Mean ± SD	1.3 ± 0.8	1.2 ± 0.8	1.5 ± 1.0	0.051
Potassium (mmol/L), Mean ± SD	3.9 ± 0.6	3.9 ± 0.5	4.0 ± 0.8	0.088
Sodium (mmol/L), Mean ± SD	137.3 ± 4.9	137.3 ± 4.5	137.4 ± 6.0	0.861
Prothrombin time (s), Median (IQR)	13.5 (12.1, 15.0)	13.3 (12.0, 14.9)	14.6 (13.0, 17.3)	0.007
Activated partial thromboplastin time (s), Mean ± SD	33.8 ± 12.7	32.7 ± 6.1	37.7 ± 24.6	0.026
Thrombin time (s), Mean ± SD	14.7 ± 3.8	14.5 ± 1.8	15.5 ± 7.4	0.129
Fibrinogen (g/L), Mean ± SD	5.3 ± 1.8	5.4 ± 1.8	4.9 ± 2.0	0.126
International normalized ratio, Median (IQR)	1.2 (1.1, 1.4)	1.2 (1.1, 1.3)	1.3 (1.2, 1.5)	0.012
Procalcitonin (ng/mL), Median (IQR)	0.3 (0.2, 0.5)	0.3 (0.2, 0.4)	0.3 (0.1, 9.6)	0.993
White blood cell count ( × 10^9^/L), Mean ± SD	12.9 ± 6.4	13.6 ± 6.4	10.6 ± 5.7	0.009
Neutrophil count ( × 10^9^/L),Mean ± SD	10.9 ± 6.0	11.4 ± 6.1	8.9 ± 5.3	0.018
Lymphocyte count ( × 10^9^/L), Mean ± SD	1.3 ± 0.8	1.4 ± 0.8	0.9 ± 0.7	0.002
Monocyte count ( × 10^9^/L), Mean ± SD	0.9 ± 0.5	1.0 ± 0.5	0.7 ± 0.7	0.011
Red Cell Distribution Width %, Mean ± SD	15.0 ± 3.7	14.9 ± 3.9	15.3 ± 2.9	0.536
Platelet count ( × 10^9^/L), Mean ± SD	262.6 ± 143.0	286.2 ± 133.5	177.5 ± 145.4	< 0.001
C-reactive protein (mg/L), Mean ± SD	134.8 ± 79.7	138.4 ± 79.6	121.8 ± 79.7	0.244
Mean platelet volume (fL), Mean ± SD	9.9 ± 1.4	9.8 ± 1.2	10.0 ± 1.9	0.437
Hemoglobin (g/L), Mean ± SD	109.7 ± 28.3	110.3 ± 27.6	107.5 ± 30.7	0.574
Hematocrit %, Mean ± SD	33.6 ± 8.4	33.9 ± 8.1	32.6 ± 9.4	0.401
Hypertension, n (%)				0.364
No	142 (77.2)	109 (75.7)	33 (82.5)	
Yes	42 (22.8)	35 (24.3)	7 (17.5)	
Diabetes, *n* (%)				0.913
No	86 (46.7)	67 (46.5)	19 (47.5)	
Yes	98 (53.3)	77 (53.5)	21 (52.5)	
Shock, *n* (%)				< 0.001
No	139 (75.5)	125 (86.8)	14 (35)	
Yes	45 (24.5)	19 (13.2)	26 (65)	
Sepsis, *n* (%)				< 0.001
No	121 (65.8)	106 (73.6)	15 (37.5)	
Yes	63 (34.2)	38 (26.4)	25 (62.5)	
Time to debridement, *n* (%)				0.007
≤ 12 h	94 (51.1)	66 (45.8)	28 (70)	
>12 h	90 (48.9)	78 (54.2)	12 (30)	
Infection site, *n* (%)				0.701
Cervical	4 (2.2)	4 (2.8)	0 (0)	
Extremity	99 (53.8)	80 (55.6)	19 (47.5)	
Multiple sites	14 (7.6)	10 (6.9)	4 (10)	
Perineal/Fournier's gangrene	38 (20.7)	28 (19.4)	10 (25)	
Trunk/abdominal wall	29 (15.8)	22 (15.3)	7 (17.5)	
LRINEC, Mean ± SD	6.3 ± 2.7	6.0 ± 2.6	7.4 ± 2.7	0.004
APACHE II, Mean ± SD	11.6 ± 6.9	11.5 ± 6.2	11.8 ± 9.0	0.772
SOFA, Mean ± SD	6.1 ± 3.9	5.9 ± 3.6	6.8 ± 4.7	0.206
DBLR, Median (IQR)	5.0 (1.9, 11.8)	3.9 (1.7, 9.9)	10.1 (5.0, 66.6)	< 0.001
log_10_(DBLR), Median (IQR)	0.7 (0.3, 1.1)	0.6 (0.2, 1.0)	1.0 (0.7, 1.8)	< 0.001

APACHE II, Acute Physiology and Chronic Health Evaluation II; CI, confidence interval; DBLR, Direct Bilirubin-to-Lymphocyte Ratio; HR, hazard ratio; IQR, Interquartile Range; LRINEC, Laboratory Risk Indicator for Necrotizing Fasciitis; SD, Standard Deviation; SOFA, Sequential Organ Failure Assessment.

The median DBLR was 5.0 (IQR, 1.9–11.8). Age, sex, and hypertension were similar across DBLR quartiles, whereas diabetes differed significantly. Patients in higher DBLR quartiles showed more severe hepatic dysfunction, coagulation abnormalities, immune-cell depletion, and inflammatory burden. These changes included lower albumin and platelet count, higher direct bilirubin and INR, prolonged prothrombin time, increased CRP, and lower lymphocyte count. Shock, sepsis, LRINEC, APACHE II, and SOFA also differed across quartiles. Mortality increased stepwise from 6.5% in Q1 to 39.1% in Q4 (Supplementary Table S1).

### 3.2 Association between DBLR and 28-day mortality

**Figure 2:**
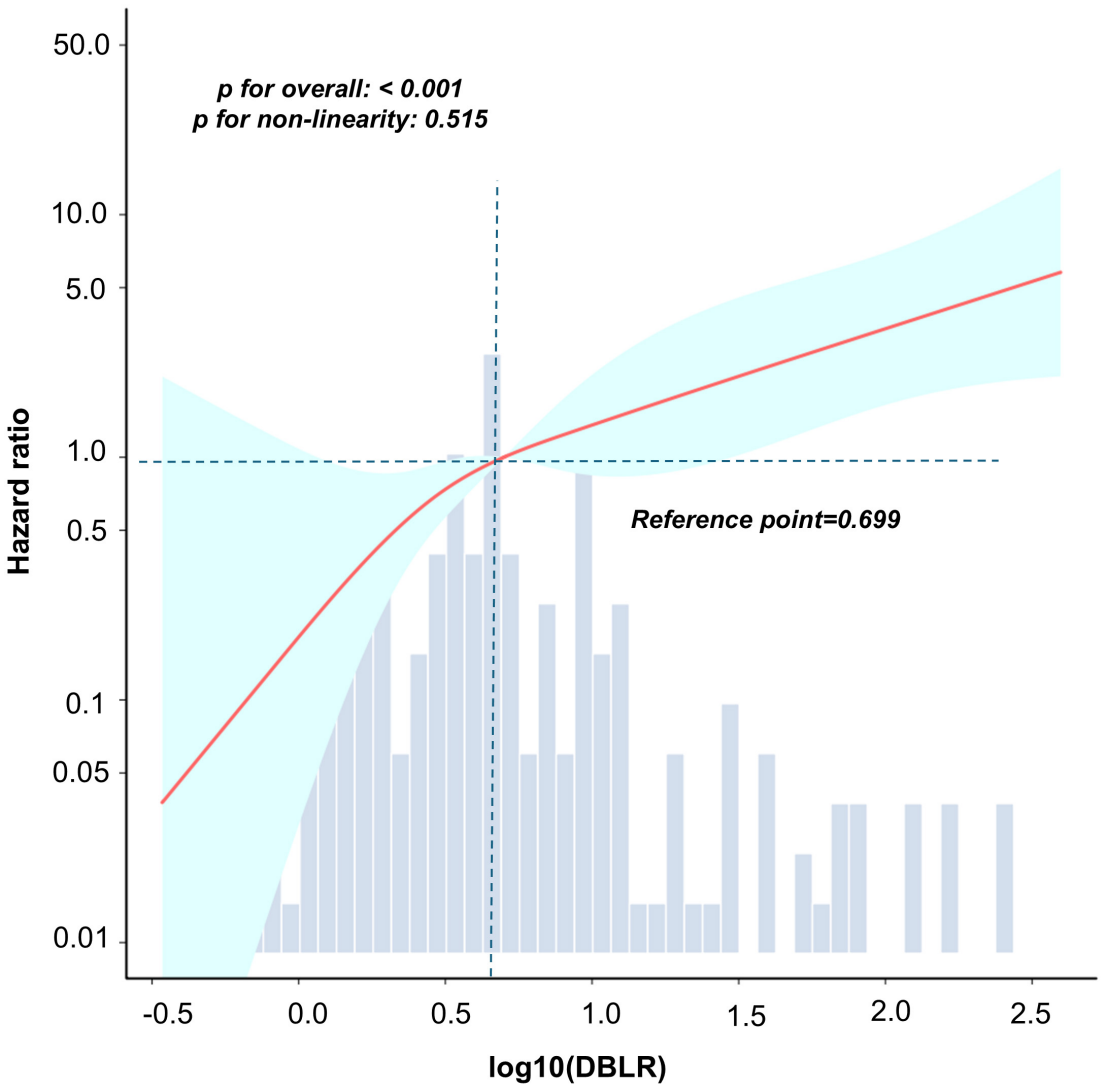
Restricted cubic spline (RCS) analysis of log_10_(DBLR) and 28-day mortality. The reference point was set at the median value of log_10_(DBLR), 0.699. RCS analysis was performed using four knots. The red line represents the estimated hazard ratio, and the shaded area represents the 95% confidence interval. The histogram shows the distribution of log_10_(DBLR). DBLR, direct bilirubin-to-lymphocyte ratio; RCS, restricted cubic spline.

RCS analysis showed that raw DBLR had a non-linear association with 28-day mortality (Supplementary Figure S2). The median raw DBLR value was 5.0. After log_10_ transformation, the association became approximately linear. The spline analysis showed a significant overall association between log_10_(DBLR) and 28-day mortality, with no evidence of non-linearity (Figure 2). The reference point was set at the median value of log_10_(DBLR), which was 0.699. RCS analysis was performed using four knots. Therefore, log_10_(DBLR) was used as the primary exposure variable in the Cox regression models.

The proportion of missing data for each candidate variable is shown in Supplementary Table S2. The variables included in the final Cox models, including log_10_(DBLR), age, shock, LRINEC, SOFA, APACHE II, survival time, and 28-day mortality status, had no missing values. Therefore, the complete-case analysis included all 184 patients and yielded the same estimates as the primary analysis.

**Figure 3:**
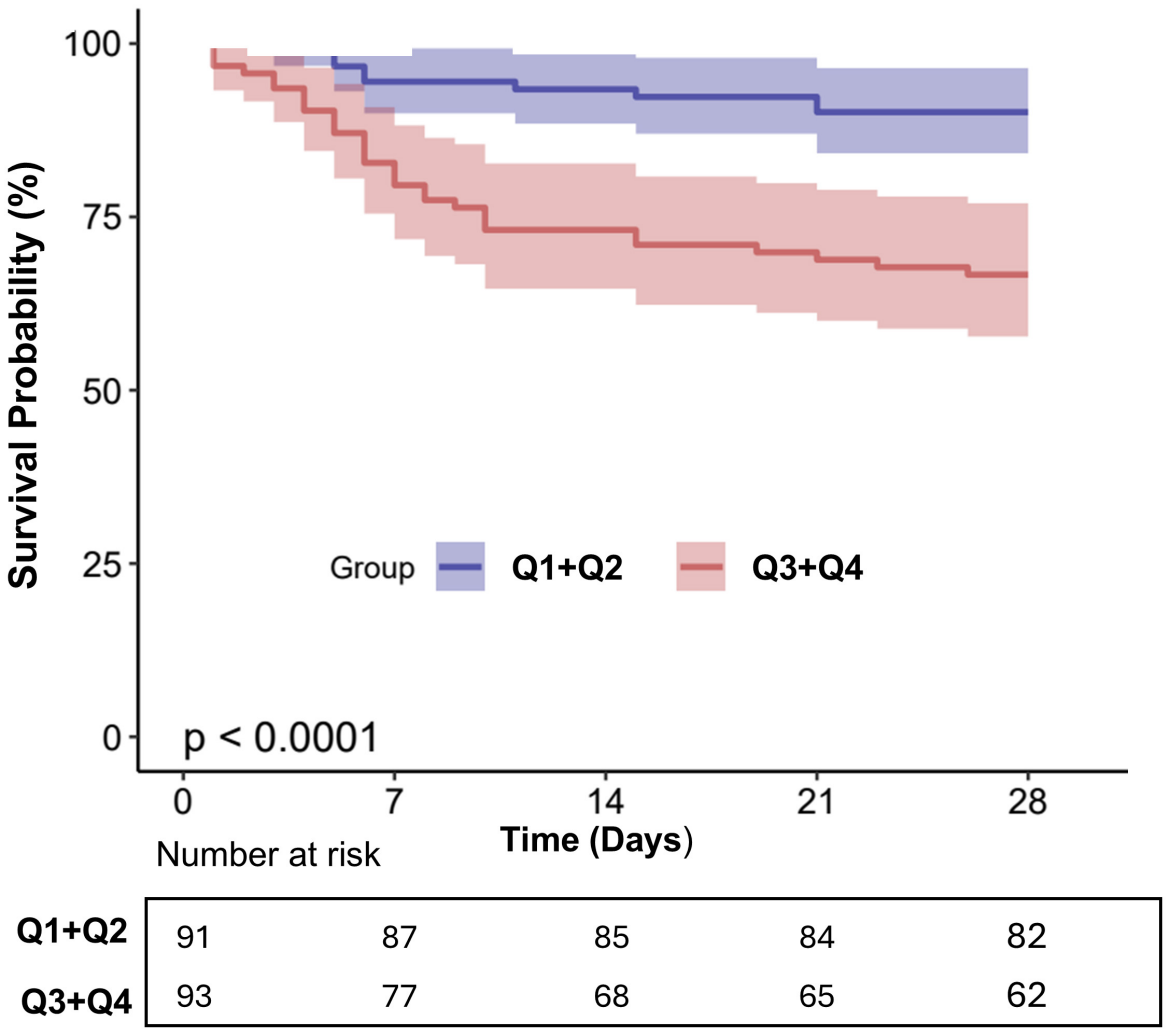
Kaplan–Meier curves for 28-day survival according to lower and higher DBLR groups. Patients were divided into lower and higher DBLR groups by collapsing quartiles into Q1+Q2 and Q3+Q4. The Q1+Q2 group represented the lower DBLR group, and the Q3+Q4 group represented the higher DBLR group. Survival differences were compared using the log-rank test (*P* < 0.0001). The Q3+Q4 group showed significantly lower 28-day survival than the Q1+Q2 group. DBLR, direct bilirubin-to-lymphocyte ratio; NF, necrotizing fasciitis.

Kaplan-Meier analysis showed lower 28-day survival in patients with higher DBLR levels. Because the number of deaths in the lowest quartile was small, the four-quartile survival analysis was interpreted as descriptive rather than confirmatory. To provide a more stable categorical comparison, DBLR quartiles were collapsed into Q1–Q2 and Q3–Q4. Patients in the Q3–Q4 group showed significantly lower 28-day survival than those in the Q1–Q2 group (log-rank *P* < 0.0001; Figure 3). This finding was consistent with the primary Cox analysis using log_10_(DBLR) as a continuous variable.

This result was also supported by the univariable Cox model. Following multivariable adjustment, the direction of the association remained consistent (Table 2). In Cox regression analysis, higher log_10_ (DBLR) was consistently associated with increased 28-day mortality. In the unadjusted model, the HR was 3.258 (95% CI, 2.143–4.952; *P* < 0.001). The association remained significant after adjustment for age, shock, and LRINEC in Model 3 (HR, 2.119; 95% CI, 1.384–3.243; *P* < 0.001). Similar associations were observed in the SOFA-adjusted model and the APACHE II-adjusted model. To address treatment-related confounding, we further adjusted for early debridement status in Model 6 as a sensitivity analysis. After additional adjustment for early debridement within 12 h vs. delayed or no debridement, log_10_(DBLR) remained associated with 28-day mortality (HR, 1.730; 95% CI, 1.049–2.854; *P* = 0.032).

**Table 2:** Cox regression analysis of the association between log_10_(DBLR) and 28-day mortality in patients with necrotizing fasciitis.

Variable	Unadjusted		Model 1		Model 2		Model 3		Model 4		Model 5		Model 6	
	HR (95% CI)	*P* value	HR (95% CI)	*P* value	HR (95% CI)	*P* value	HR (95% CI)	*P* value	HR (95% CI)	*P* value	HR (95% CI)	*P* value	HR (95% CI)	*P* value
log_10_(DBLR)	3.258 (2.143–4.952)	< 0.001	3.241 (2.124–4.947)	< 0.001	2.232 (1.474–3.380)	< 0.001	2.119 (1.384–3.243)	< 0.001	3.215 (2.076–4.980)	< 0.001	3.381 (2.171–5.264)	< 0.001	1.730 (1.049–2.854)	0.032

APACHE II, Acute Physiology and Chronic Health Evaluation II; CI, confidence interval; DBLR, Direct Bilirubin-to-Lymphocyte Ratio; HR, hazard ratio; LRINEC, Laboratory Risk Indicator for Necrotizing Fasciitis; SOFA, Sequential Organ Failure Assessment.

Model 1 was adjusted for age.

Model 2 was further adjusted for shock.

Model 3 was further adjusted for LRINEC.

Model 4 was adjusted for SOFA.

Model 5 was adjusted for APACHE II.

Model 6 was based on Model 3 and further adjusted for early debridement within 12 h vs. delayed or no debridement.

### 3.3 Subgroup analysis

**Figure 4:**
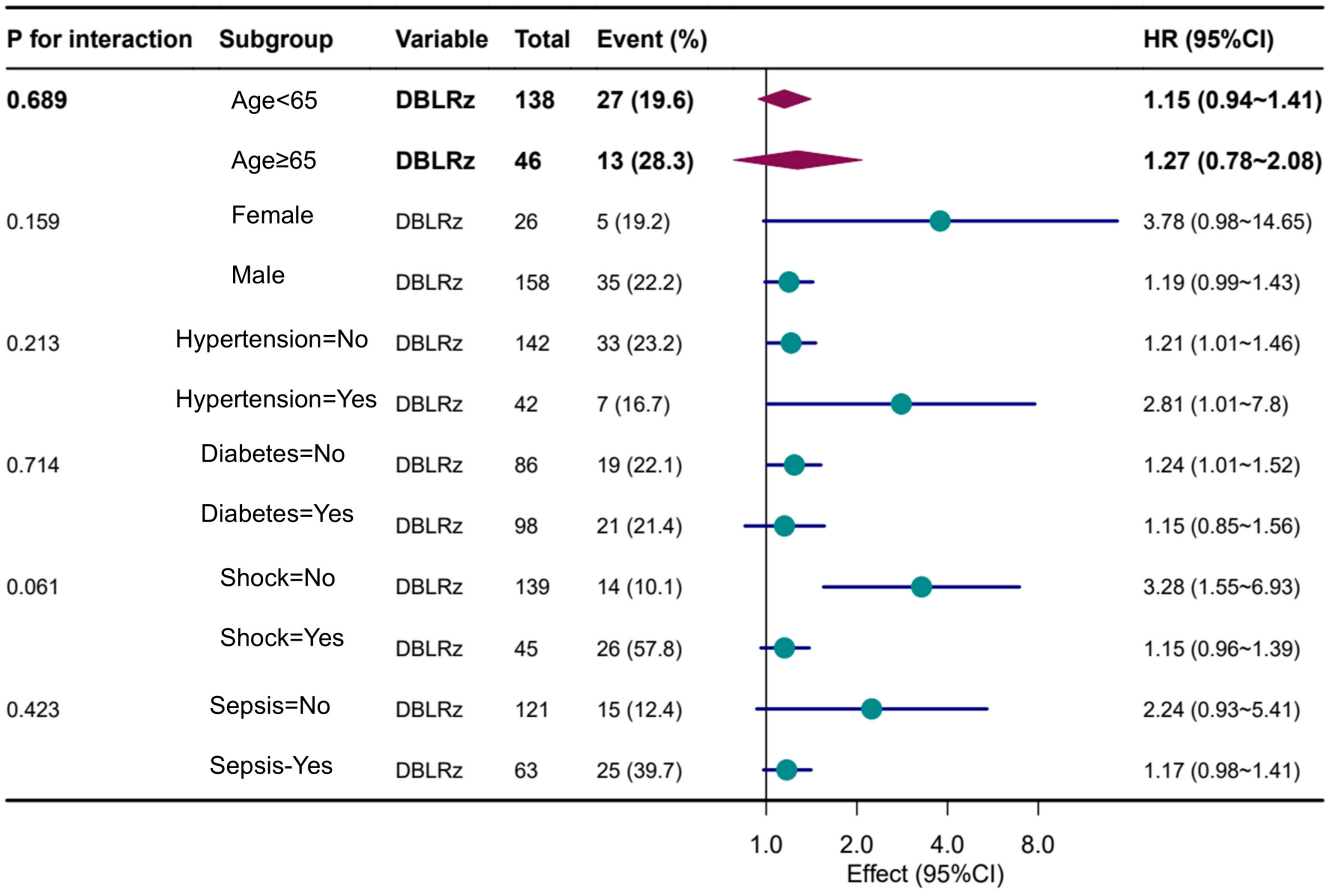
Subgroup analyses assessing the association between z-standardized DBLR (DBLRz) and 28-day mortality across predefined clinical strata. DBLR was z-standardized as DBLRz = (DBLR - mean) / SD. Hazard ratios represent the change in mortality risk per one standard deviation increase in DBLR. Diamonds and squares indicate the HR point estimate with 95% CIs for each subgroup. *P*-values for interaction are listed on the left. CI, confidence interval; DBLR, direct bilirubin-to-lymphocyte ratio; HR, hazard ratio.

The subgroup analysis is shown in Figure 4. Overall, the association between DBLR and 28-day mortality showed a generally consistent direction across prespecified clinical subgroups. The subgroup-specific HRs per standard deviation increase in DBLRz were 1.15 (95% CI, 0.94–1.41) in patients aged < 65 years and 1.27 (95% CI, 0.78–2.08) in patients aged ≥65 years. The corresponding HRs were 3.78 (95% CI, 0.98–14.65) in female patients and 1.19 (95% CI, 0.99–1.43) in male patients. In patients without and with hypertension, the HRs were 1.21 (95% CI, 1.01–1.46) and 2.81 (95% CI, 1.00–7.80), respectively. In patients without and with diabetes, the HRs were 1.24 (95% CI, 1.00–1.52) and 1.15 (95% CI, 0.85–1.56), respectively. In patients without and with shock, the HRs were 3.28 (95% CI, 1.55–6.93) and 1.15 (95% CI, 0.96–1.39), respectively. In patients without and with sepsis, the HRs were 2.24 (95% CI, 0.93–5.41) and 1.17 (95% CI, 0.98–1.41), respectively.

No interaction reached conventional statistical significance. However, the interaction by shock status was borderline (*P* for interaction = 0.061), suggesting a possible difference in the DBLR-mortality association according to shock status. Given the limited number of deaths within several subgroups, these subgroup analyses were considered exploratory.

### 3.4 Predictive performance

**Figure 5:**
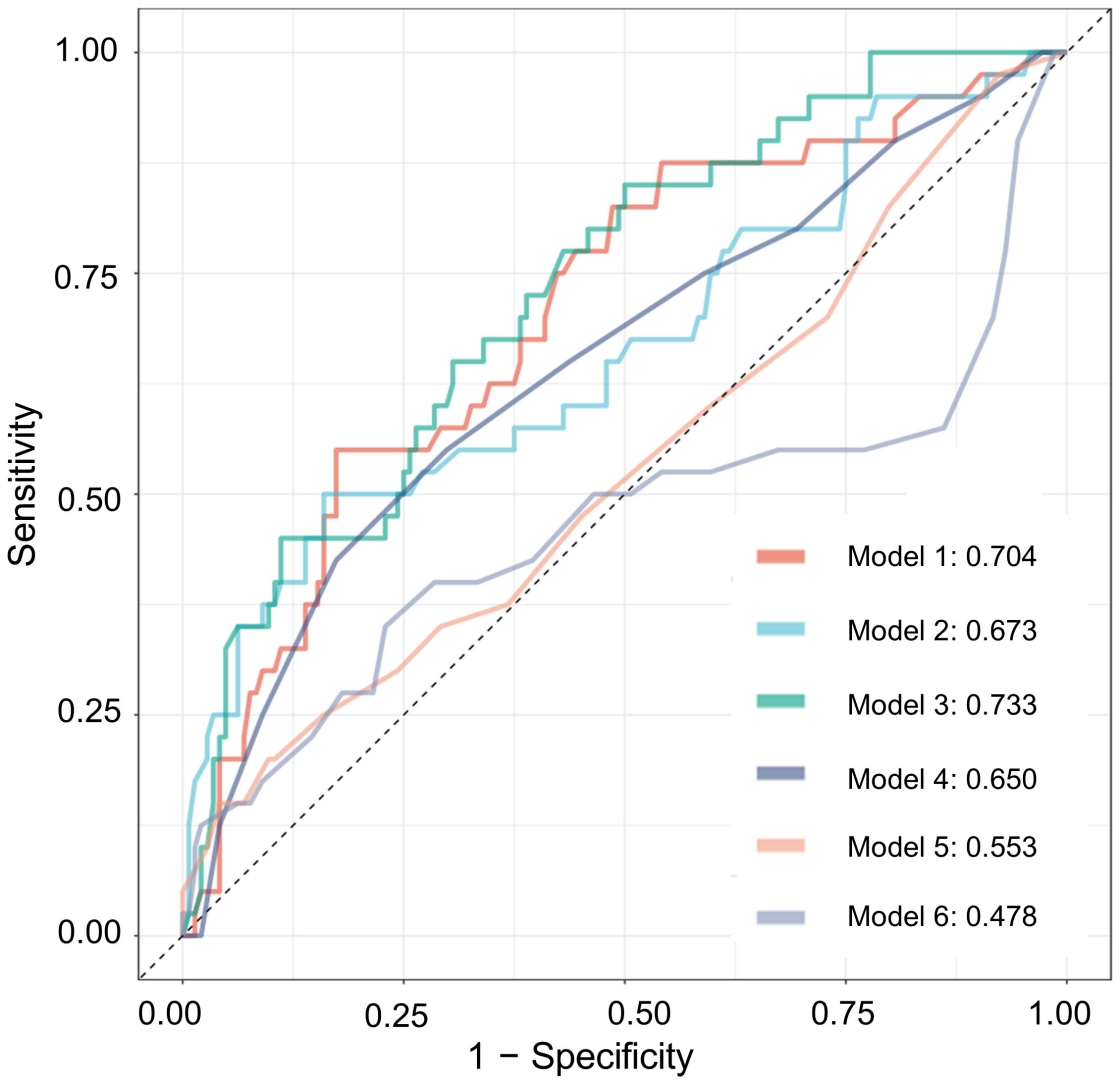
ROC curves for predicting 28-day mortality in NF patients. Model 1: direct bilirubin alone, AUC = 0.704 (95% CI 0.613–0.787); Model 2: lymphocyte count alone, AUC = 0.673 (95% CI 0.571–0.762); Model 3: DBLR, AUC = 0.733 (95% CI 0.644–0.811); Model 4: LRINEC score, AUC = 0.650 (95% CI 0.545–0.741); Model 5: SOFA score, AUC = 0.553 (95% CI 0.451–0.653); Model 6: APACHE II score, AUC = 0.478 (95% CI 0.360–0.585). AUC, area under the curve; APACHE II, Acute Physiology and Chronic Health Evaluation II; DBLR, direct bilirubin-to-lymphocyte ratio; LRINEC, Laboratory Risk Indicator for Necrotizing Fasciitis; NF, necrotizing fasciitis; ROC, receiver operating characteristic; SOFA, Sequential Organ Failure Assessment.

ROC analysis showed that DBLR had the highest AUC among the evaluated markers and scores, although its discrimination was modest-to-acceptable rather than strong (AUC, 0.733; 95% CI, 0.644–0.811). The AUCs for direct bilirubin, lymphocyte count, LRINEC, SOFA, and APACHE II were 0.704, 0.673, 0.650, 0.553, and 0.478, respectively (Figure 5).

To further characterize the clinical profile reflected by DBLR, a correlation heatmap of selected variables was generated using Spearman's rank correlation analysis (Supplementary Figure S2). DBLR showed a strong positive correlation with direct bilirubin (r = 0.75) and a strong inverse correlation with lymphocyte count (r = −0.75), consistent with its mathematical composition. In addition, DBLR was positively correlated with prothrombin time, LRINEC, shock, sepsis, and 28-day mortality, whereas an inverse correlation was observed with albumin. Overall, these findings suggest that higher DBLR values tend to cluster with greater disease severity, coagulation disturbance, and unfavorable short-term outcomes in patients with necrotizing fasciitis.

## 4 Discussion

The main finding of this study was that DBLR was associated with 28-day all-cause mortality in patients with NF. After log_10_ transformation, DBLR remained associated with mortality in Cox regression models. The survival analysis also showed lower 28-day survival among patients with higher DBLR levels. However, categorical comparisons based on DBLR quartiles should be interpreted cautiously because the number of deaths in the lowest quartile was small. Therefore, quartile-based results were treated as secondary descriptive findings, while the continuous log_10_(DBLR) model was used as the primary analysis.

The mechanistic basis for DBLR as a prognostic composite rests on the distinct yet interrelated pathophysiology of its constituents. Direct bilirubin elevation in NF reflects hepatocyte injury and biliary dysfunction propagated by the same pathological cascade driving tissue necrosis: massive pro-inflammatory cytokine release (interleukin-1β, tumor necrosis factor-α, interleukin-6), microvascular thrombosis, and hepatic ischaemia-reperfusion injury. In patients progressing to septic shock, hepatic hypoperfusion and cholestasis further amplify circulating direct bilirubin. This was corroborated by the substantially higher DBLR values and markedly greater prevalence of shock (50.0% vs. 15.2% in Q1) recorded in Q4 patients, who also exhibited higher SOFA scores. Hypoalbuminemia, present to a greater degree in non-survivors in this cohort, is known to reflect both hepatic synthetic failure and the redistribution of albumin during systemic inflammation (25, 30). Conversely, lymphocytopenia in severe NF arises principally from accelerated apoptosis driven by the sepsis-associated cytokine storm, regulatory T-cell expansion, and cortisol-mediated lymphocyte redistribution (15, 21). Sustained lymphocytopenia signals immunological exhaustion, a state in which adaptive immunity can no longer contain infection, and has been linked to adverse outcomes spanning sepsis to peritonitis (15, 25). DBLR combines liver dysfunction and immune cell depletion into a single ratio, capturing a pattern of injury that neither direct bilirubin nor lymphocyte count can reveal on its own. This biological completeness underpins the enhanced prognostic discrimination observed with DBLR compared to its individual components.

It is helpful to compare DBLR to well-known prognostic indicators. Wu et al. found that NLR and PLR, the two most widely verified hematological ratios in NF, could both predict death on their own with AUCs of 0.790 and 0.679, respectively (28). These results have been confirmed in additional comparative analyses (31). The current DBLR AUC of 0.733 is higher than PLR and close to NLR. It also includes liver information that is not seen in solely hematological composites. The LRINEC score was first designed to help with diagnostic triage, not prognostic classification (26). In this group, it had an AUC of 0.650 for 28-day mortality, which is in line with growing evidence that it may not be useful for predicting outcomes (18, 32). In the work of Xu et al. (16), CAR and SIRI were shown to be useful for predicting the severity and occurrence of NF on their own. The current results suggest that DBLR looks at a different biological aspect, specifically liver dysfunction, and may therefore add to these indices instead of repeating them. The RAR that Kayar et al. (17) reported had an impressive AUC of 0.955 in scrotal Fournier's gangrene. However, that group was limited to a single anatomical variant (*n* = 62), which makes it harder to apply to the wider NF spectrum. However, the prognostic value of RAR has been shown to be true in populations with sepsis and bacteremia (13, 22, 29). The anatomically diverse group in this study may give a better picture of how useful DBLR is for predicting outcomes.

The correlation heatmap provided supportive context for the prognostic role of DBLR in this cohort. In addition to its expected relationships with direct bilirubin and lymphocyte count, DBLR also showed associations with albumin, prothrombin time, LRINEC, shock, sepsis, and 28-day mortality. This pattern suggests that DBLR may reflect a broader state of systemic deterioration involving hepatobiliary dysfunction, immune depletion, coagulation abnormality, and overall clinical severity, rather than representing an isolated laboratory abnormality. Although correlation does not imply causation, these findings offer additional biological and clinical support for the observed association between DBLR and mortality.

DBLR has practical clinical advantages. It can be computed immediately from indices routinely obtained at emergency admission, imposing no additional cost or procedural burden, and is therefore applicable at the point of first contact. The non-linear pattern of raw DBLR in spline analysis was attributable to its right-skewed distribution and sparse extreme-value observations; log10 transformation stabilized the modeling scale and was therefore adopted for all primary analyses, with the raw-scale spline retained as a supplementary reference. Subgroup analyses should be interpreted cautiously: although no interaction reached statistical significance, the borderline shock-subgroup interaction signal may reflect biological heterogeneity, differential baseline severity, or limited statistical power after stratification. This finding is exploratory and requires confirmation in larger, independent cohorts. Admission-level predictors of mortality in NF have previously encompassed hemodynamic instability, renal dysfunction, and extent of soft-tissue involvement (17, 20); DBLR may capture an additional dimension, namely hepatic metabolic compromise, not fully reflected in these parameters. That this association persisted following adjustment for both shock and LRINEC further implies that DBLR captures prognostic signal not fully encoded by existing clinical parameters.

Several limitations warrant acknowledgment. First, the single-center design limits the generalizability of our findings. All patients were treated at a regional hospital in Guangxi, China, and the cohort had a specific demographic and clinical case-mix. External validation was not available in the present study. Therefore, future studies should validate DBLR in independent multicenter cohorts before clinical implementation. Recent scoring systems, SIARI, J-LRINEC, and the NEJM algorithm are important diagnostic frameworks for suspected necrotizing fasciitis, particularly when differentiating NF from cellulitis or other soft-tissue infections (4, 23, 33). However, they were not used as direct comparison benchmarks in the present study because our cohort included only confirmed NF cases and the primary outcome was 28-day mortality rather than diagnostic discrimination. In addition, BMI and immunodeficiency status were not consistently available in the retrospective records, which prevented reliable calculation of SIARI or J-LRINEC. Future prospective studies should collect all required components and evaluate DBLR together with these diagnostic frameworks. Second, the relatively modest non-survivor count (*n* = 40), whilst adequate to detect the observed associations, constrains subgroup estimate precision and the stability of high-dimensional regression models, as evidenced by the wide confidence intervals in Q4. Finally, the decade-long study window encompasses potential secular changes in management protocols (6, 7), and temporal confounding was not formally addressed.

## 5 Conclusion

In conclusion, DBLR was associated with 28-day mortality in patients with necrotizing fasciitis in this single-center retrospective cohort from Guangxi, China. After log_10_ transformation, DBLR showed a stable association with mortality and may serve as a simple admission biomarker for early risk stratification. However, because this cohort came from a single regional hospital with specific demographic and clinical characteristics, the generalizability of these findings remains limited. Future multicenter studies and independent external validation cohorts are needed before DBLR can be used routinely in clinical practice.

## Data Availability

The original contributions presented in the study are included in the article/Supplementary material, further inquiries can be directed to the corresponding author.
