# Supplementary material for: Association between the direct bilirubin to lymphocyte ratio and mortality in patients with necrotizing fasciitis: a retrospective cohort study
Source: Front Med (Lausanne). 2026 Jun 1;13:1841323. doi: 10.3389/fmed.2026.1841323 (PMC13267103; doi:10.3389/fmed.2026.1841323)
Supplement: Supplementary file 1 [file Supplementary_file_1.docx]

## Supplementary Tables

**Supplementary Table 1**. Baseline characteristics of patients categorized by DBLR quartile.

| Variables | Total (n = 184) | Q1 (DBLR<1.936) (n = 46) | Q2 (1.936≤DBLR<5.000) (n = 45) | Q3 (5.000≤DBLR<11.615) (n = 47) | Q4 (DBLR≥11.615) (n = 46) | P |
| --- | --- | --- | --- | --- | --- | --- |
| Age (years), Mean ± SD | 57.054 ± 11.921 | 56.435 ± 11.916 | 56.000 ± 10.097 | 58.894 ± 13.065 | 56.826 ± 12.515 | 0.660 |
| Gender, n (%) |  |  |  |  |  | 0.627 |
| Female | 26 (14.130) | 7 (15.217) | 7 (15.556) | 4 (8.511) | 8 (17.391) |  |
| Male | 158 (85.870) | 39 (84.783) | 38 (84.444) | 43 (91.489) | 38 (82.609) |  |
| Urea (mmol/L), Median (IQR) | 6.555 (4.553, 12.230) | 5.195 (4.372, 7.463) | 5.710 (4.310, 8.000) | 6.720 (5.180, 12.440) | 10.580 (6.352, 16.645) | < 0.001 |
| Creatinine (µmol/L), Median (IQR) | 82.000 (64.000, 129.000) | 73.000 (60.250, 103.000) | 78.000 (64.000, 99.000) | 86.000 (70.000, 132.500) | 94.000 (63.250, 160.000) | 0.144 |
| Uric acid (µmol/L), Mean ± SD | 343.351 ± 162.880 | 296.650 ± 127.870 | 327.845 ± 147.555 | 353.948 ± 141.557 | 394.394 ± 210.986 | 0.029 |
| Total Protein (g/L), Mean ± SD | 58.629 ± 10.223 | 62.350 ± 7.789 | 61.876 ± 8.752 | 60.094 ± 10.383 | 50.237 ± 8.962 | < 0.001 |
| Albumin (g/L), Mean ± SD | 28.349 ± 6.729 | 30.554 ± 5.007 | 31.269 ± 7.090 | 29.115 ± 6.079 | 22.507 ± 4.808 | < 0.001 |
| Total bilirubin (µmol/L), Median (IQR) | 11.850 (7.000, 20.875) | 5.750 (4.350, 6.925) | 9.100 (7.300, 12.900) | 14.900 (11.150, 21.850) | 27.250 (17.700, 47.500) | < 0.001 |
| Direct bilirubin (µmol/L), Median (IQR) | 5.050 (2.875, 9.925) | 2.200 (1.600, 2.700) | 3.800 (3.000, 4.800) | 7.300 (5.600, 10.050) | 18.550 (9.400, 30.625) | < 0.001 |
| Indirect bilirubin (µmol/L), Median (IQR) | 5.600 (3.400, 9.525) | 3.400 (2.300, 4.200) | 5.500 (3.200, 7.900) | 6.200 (3.850, 10.100) | 9.900 (8.000, 15.350) | < 0.001 |
| Globulin (g/L), Mean ± SD | 30.542 ± 7.990 | 32.685 ± 10.455 | 30.402 ± 5.967 | 30.821 ± 7.224 | 28.252 ± 7.211 | 0.066 |
| Aspartate Aminotransferase (U/L), Median (IQR) | 25.000 (16.875, 45.250) | 18.500 (13.250, 27.000) | 23.000 (15.000, 28.000) | 25.000 (19.500, 49.500) | 46.500 (37.250, 73.500) | < 0.001 |
| Alanine Aminotransferase (U/L), Median (IQR) | 26.000 (15.000, 47.000) | 22.450 (15.250, 41.000) | 23.000 (14.000, 51.000) | 30.000 (14.500, 52.500) | 32.500 (19.925, 47.000) | 0.246 |
| AST/ALT, Mean ± SD | 1.263 ± 0.829 | 0.873 ± 0.381 | 1.031 ± 0.586 | 1.299 ± 0.809 | 1.842 ± 1.043 | < 0.001 |
| Potassium (mmol/L), Mean ± SD | 3.907 ± 0.594 | 3.881 ± 0.343 | 3.870 ± 0.529 | 3.966 ± 0.652 | 3.908 ± 0.777 | 0.87 |
| Sodium(mmol/L), Mean ± SD | 137.302 ± 4.871 | 138.696 ± 4.849 | 137.266 ± 2.942 | 135.649 ± 5.014 | 137.633 ± 5.820 | 0.023 |
| Prothrombin Time (s), Median (IQR) | 13.470 (12.075, 15.000) | 12.050 (11.000, 13.560) | 12.800 (12.000, 14.100) | 14.600 (13.210, 15.750) | 14.300 (13.225, 17.375) | < 0.001 |
| Activated Partial Thromboplastin Time (s), Mean ± SD | 33.777 ± 12.743 | 30.599 ± 3.871 | 31.962 ± 5.196 | 33.211 ± 5.670 | 39.307 ± 23.258 | 0.005 |
| Thrombin Time (s), Mean ± SD | 14.736 ± 3.759 | 14.577 ± 1.375 | 14.391 ± 2.124 | 14.419 ± 2.083 | 15.555 ± 6.768 | 0.398 |
| Fibrinogen (g/L), Mean ± SD | 5.293 ± 1.825 | 5.091 ± 1.556 | 5.445 ± 1.553 | 5.635 ± 2.340 | 4.996 ± 1.702 | 0.292 |
| International Normalized Ratio, Median (IQR) | 1.220 (1.110, 1.355) | 1.120 (1.020, 1.212) | 1.170 (1.090, 1.260) | 1.300 (1.195, 1.405) | 1.292 (1.200, 1.522) | < 0.001 |
| Procalcitonin (ng/mL), Median (IQR) | 0.320 (0.208, 0.520) | 0.345 (0.260, 0.410) | 0.310 (0.210, 0.410) | 0.265 (0.193, 0.415) | 0.465 (0.192, 17.730) | 0.122 |
| White Blood Cell count (×10^9^/L), Mean ± SD | 12.939 ± 6.391 | 13.371 ± 6.182 | 12.916 ± 6.357 | 14.350 ± 6.506 | 11.089 ± 6.271 | 0.094 |
| Neutrophil count (×10^9^/L), Mean ± SD | 10.888 ± 6.017 | 10.208 ± 5.681 | 10.640 ± 6.045 | 12.595 ± 6.132 | 10.066 ± 6.042 | 0.15 |
| Lymphocyte count (×10^9^/L), Mean ± SD | 1.276 ± 0.809 | 2.017 ± 0.677 | 1.270 ± 0.510 | 1.196 ± 0.707 | 0.623 ± 0.662 | < 0.001 |
| Monocyte count (×10^9^/L), Mean ± SD | 0.866 ± 0.463 | 1.099 ± 0.502 | 0.883 ± 0.386 | 0.915 ± 0.398 | 0.566 ± 0.402 | < 0.001 |
| Red Cell Distribution Width %, Mean ± SD | 15.001 ± 3.731 | 14.276 ± 4.167 | 14.898 ± 4.693 | 14.866 ± 2.699 | 15.963 ± 2.959 | 0.18 |
| Platelet count (×10^9^/L), Mean ± SD | 262.609 ± 142.991 | 355.587 ± 132.574 | 284.733 ± 123.359 | 256.234 ± 130.975 | 154.500 ± 108.820 | < 0.001 |
| C-reactive protein (mg/L), Mean ± SD | 130.897 ± 68.774 | 99.847 ± 71.413 | 120.781 ± 66.410 | 138.336 ± 65.474 | 164.243 ± 56.198 | < 0.001 |
| Mean platelet volume (fL), Mean ± SD | 9.874 ± 1.391 | 9.872 ± 0.886 | 9.488 ± 1.604 | 9.863 ± 1.142 | 10.263 ± 1.713 | 0.069 |
| Hemoglobin (g/L), Mean ± SD | 109.728 ± 28.252 | 112.326 ± 20.392 | 109.133 ± 28.727 | 110.319 ± 32.296 | 107.109 ± 30.698 | 0.846 |
| Hematocrit %, Mean ± SD | 33.577 ± 8.393 | 34.913 ± 6.125 | 33.529 ± 8.525 | 33.668 ± 9.563 | 32.196 ± 8.977 | 0.493 |
| Hypertension, n (%) |  |  |  |  |  | 0.503 |
| No | 142 (77.174) | 38 (82.609) | 32 (71.111) | 38 (80.851) | 34 (73.913) |  |
| Yes | 42 (22.826) | 8 (17.391) | 13 (28.889) | 9 (19.149) | 12 (26.087) |  |
| Diabetes, n (%) |  |  |  |  |  | 0.015 |
| No | 86 (46.739) | 17 (36.957) | 24 (53.333) | 16 (34.043) | 29 (63.043) |  |
| Yes | 98 (53.261) | 29 (63.043) | 21 (46.667) | 31 (65.957) | 17 (36.957) |  |
| Shock, n (%) |  |  |  |  |  | < 0.001 |
| No | 138 (75.000) | 39 (84.783) | 41 (91.111) | 35 (74.468) | 23 (50) |  |
| Yes | 46 (25.000) | 7 (15.217) | 4 (8.889) | 12 (25.532) | 23 (50) |  |
| Sepsis, n (%) |  |  |  |  |  | 0.004 |
| No | 121 (65.761) | 37 (80.435) | 32 (71.111) | 31 (65.957) | 21 (45.652) |  |
| Yes | 63 (34.239) | 9 (19.565) | 13 (28.889) | 16 (34.043) | 25 (54.348) |  |
| LRINEC, Mean ± SD | 6.326 ± 2.666 | 5.739 ± 2.736 | 5.422 ± 2.398 | 6.574 ± 2.733 | 7.543 ± 2.326 | < 0.001 |
| APACHE II, Mean ± SD | 11.571 ± 6.866 | 11.696 ± 6.142 | 9.289 ± 6.048 | 11.000 ± 6.856 | 14.261 ± 7.564 | 0.005 |
| SOFA, Mean ± SD | 6.092 ± 3.851 | 6.109 ± 3.560 | 4.511 ± 3.027 | 6.191 ± 4.036 | 7.522 ± 4.173 | 0.002 |
| Time.to.debridement, n (%) |  |  |  |  |  | 0.001 |
| No | 94 (51.087) | 13 (28.261) | 24 (53.333) | 25 (53.191) | 32 (69.565) |  |
| Yes | 90 (48.913) | 33 (71.739) | 21 (46.667) | 22 (46.809) | 14 (30.435) |  |
| Infection.site, n (%) |  |  |  |  |  | 0.318 |
| Cervical | 4 ( 2.174) | 2 (4.348) | 2 (4.444) | 0 (0) | 0 (0) |  |
| Extremity | 99 (53.804) | 22 (47.826) | 22 (48.889) | 26 (55.319) | 29 (63.043) |  |
| Multiple sites | 14 ( 7.609) | 1 (2.174) | 4 (8.889) | 5 (10.638) | 4 (8.696) |  |
| Perineal/Fournier’s gangrene | 38 (20.652) | 10 (21.739) | 10 (22.222) | 12 (25.532) | 6 (13.043) |  |
| Trunk/abdominal wall | 29 (15.761) | 11 (23.913) | 7 (15.556) | 4 (8.511) | 7 (15.217) |  |
| Mortality, n (%) |  |  |  |  |  | < 0.001 |
| Survivors | 144 (78.261) | 43 (93.478) | 39 (86.667) | 34 (72.340) | 28 (60.870) |  |
| Non-survivors | 40 (21.739) | 3 (6.522) | 6 (13.333) | 13 (27.660) | 18 (39.130) |  |
| DBLR, Median (IQR) | 5.000 (1.933, 11.792) | 1.191 (0.834, 1.621) | 3.235 (2.667, 3.919) | 7.692 (5.503, 9.710) | 40.686 (19.619, 89.109) | < 0.001 |
| log10(DBLR), Median (IQR) | 0.699 (0.286, 1.071) | 0.076 (-0.079, 0.210) | 0.510 (0.426, 0.593) | 0.886 (0.741, 0.987) | 1.609 (1.292, 1.950) | < 0.001 |

Note: DBLR, Direct Bilirubin-to-Lymphocyte Ratio; SD, Standard Deviation; IQR, Interquartile Range; LRINEC, Laboratory Risk Indicator for Necrotizing Fasciitis; SOFA, Sequential Organ Failure Assessment;APACHE II, Acute Physiology and Chronic Health Evaluation II.

**Supplementary Table 2.**  Proportion of missing data for candidate variables.

| Variables | Missing, n (%) | Handling |
| --- | --- | --- |
| Age (years) | 0, (0.0) | Complete |
| Gender | 0, (0.0) | Complete |
| Urea (mmol/L) | 1, (0.5) | Multiple imputation |
| Creatinine (µmol/L) | 0, (0.0) | Complete |
| Uric acid (µmol/L) | 8, (4.3) | Multiple imputation |
| Total Protein (g/L) | 0, (0.0) | Complete |
| Albumin (g/L) | 0, (0.0) | Complete |
| Total bilirubin (µmol/L) | 0, (0.0) | Complete |
| Direct bilirubin (µmol/L) | 0, (0.0) | Complete |
| Indirect bilirubin (µmol/L) | 0, (0.0) | Complete |
| Globulin (g/L) | 0, (0.0) | Complete |
| Aspartate Aminotransferase | 0, (0.0) | Complete |
| Alanine Aminotransferase | 0, (0.0) | Complete |
| AST/ALT Ratio | 0, (0.0) | Complete |
| Potassium (mmol/L) | 7, (3.8) | Multiple imputation |
| Sodium(mmol/L) | 7, (3.8) | Multiple imputation |
| Prothrombin Time (s) | 10, (5.4) | Multiple imputation |
| Activated Partial Thromboplastin Time (s) | 10, (5.4) | Multiple imputation |
| Thrombin Time (s) | 10, (5.4) | Multiple imputation |
| Fibrinogen (g/L) | 10, (5.4) | Multiple imputation |
| International Normalized Ratio | 10, (5.4) | Multiple imputation |
| Procalcitonin (ng/mL) | 17, (9.2) | Multiple imputation |
| White Blood Cell count (×10^9^/L) | 0, (0.0) | Complete |
| Neutrophil count (×10^9^/L) | 0, (0.0) | Complete |
| Lymphocyte count (×10^9^/L) | 0, (0.0) | Complete |
| Monocyte count (×10^9^/L) | 0, (0.0) | Complete |
| Red Cell Distribution Width % | 0, (0.0) | Complete |
| Platelet count (×10^9^/L) | 1, (0.5) | Multiple imputation |
| C-reactive protein (mg/L) | 3, (1.6) | Multiple imputation |
| Mean platelet volume (fL) | 8, (4.3) | Multiple imputation |
| Hemoglobin (g/L) | 0, (0.0) | Complete |
| Hematocrit % | 0, (0.0) | Complete |
| Hypertension | 0, (0.0) | Complete |
| Diabetes | 0, (0.0) | Complete |
| Shock | 0, (0.0) | Multiple imputation |
| Sepsis | 0, (0.0) | Complete |
| LRINEC | 0, (0.0) | Complete |
| APACHE II | 0, (0.0) | Complete |
| SOFA | 0, (0.0) | Complete |
| Mortality | 0, (0.0) | Complete |
| Time to debridement | 4, (2.2) | Multiple imputation |
| Infection site | 0, (0.0) | Complete |
| LDH | 70, (38.0) | Excluded |
| D-Dimer | 95, (51.6) | Excluded |
| CK | 83, (45.1) | Excluded |
| BNP | 82, (44.6) | Excluded |
| IL-6 | 87, (47.2) | Excluded |
| Lactate | 91, (49.5) | Excluded |

Note: DBLR, Direct Bilirubin-to-Lymphocyte Ratio; HR, hazard ratio; CI, confidence interval; LRINEC, Laboratory Risk Indicator for Necrotizing Fasciitis; SOFA, Sequential Organ Failure Assessment;APACHE II, Acute Physiology and Chronic Health Evaluation II; ICU, Intensive Care Unit; IL-6, Interleukin-6; BNP, CK, Creatine Kinase; B-type Natriuretic Peptide; LDH, Lactate Dehydrogenase.

**Supplementary Table 3.** Univariable Cox regression analysis of factors associated with 28-day mortality in patients with necrotizing fasciitis.

| Item | HR(95%CI) | P (Wald's test) |
| --- | --- | --- |
| Age (years) | 1.015 (0.989,1.041) | 0.267 |
| Gender: Male vs Female | 1.144 (0.448,2.919) | 0.779 |
| Urea (mmol/L) | 1.037 (1.013,1.063) | 0.003 |
| Creatinine (µmol/L) | 1.001 (0.999,1.002) | 0.625 |
| Uric acid (µmol/L) | 1.002 (0.999,1.003) | 0.080 |
| Total Protein (g/L) | 0.940 (0.912,0.968) | < 0.001 |
| Albumin (g/L) | 0.925 (0.881,0.972) | 0.002 |
| Total bilirubin (µmol/L) | 1.009 (1.002,1.016) | 0.016 |
| Direct bilirubin (µmol/L) | 1.012 (1.002,1.022) | 0.015 |
| Indirect bilirubin (µmol/L) | 1.030 (1.002,1.058) | 0.033 |
| Globulin (g/L) | 0.915 (0.872,0.96) | < 0.001 |
| Aspartate Aminotransferase (U/L) | 1.001 (0.998,1.004) | 0.484 |
| Alanine Aminotransferase (U/L) | 1.001 (0.996,1.006) | 0.738 |
| AST/ALT | 1.385 (1.025,1.872) | 0.034 |
| Potassium (mmol/L) | 1.587 (0.964,2.612) | 0.069 |
| Sodium(mmol/L) | 1.008(0.943,1.0769) | 0.820 |
| Prothrombin Time (s) | 1.015 (0.998,1.032) | 0.080 |
| Activated Partial Thromboplastin Time (s) | 1.035 (1.018,1.051) | < 0.001 |
| Thrombin Time (s) | 1.105 (1.04,1.173) | 0.001 |
| Fibrinogen (g/L) | 0.859 (0.712,1.036) | 0.112 |
| International Normalized Ratio | 0.974 (0.858,1.106) | 0.685 |
| Procalcitonin (ng/mL) | 1.008 (1.002,1.014) | 0.010 |
| White Blood Cell count (×109/L) | 0.919 (0.865,0.977) | 0.007 |
| Neutrophil count (×109/L) | 0.924 (0.868,0.984) | 0.013 |
| Lymphocyte count (×109/L) | 0.410 (0.238,0.707) | 0.001 |
| Monocyte count (×109/L) | 0.324 (0.147,0.711) | 0.005 |
| Red Cell Distribution Width % | 1.022 (0.955,1.094) | 0.524 |
| Platelet count (×10^9^/L) | 0.994 (0.991,0.996) | < 0.001 |
| C-reactive protein (mg/L) | 0.998 (0.994,1.002) | 0.285 |
| Mean platelet volume (fL) | 1.145 (0.857,1.528) | 0.359 |
| Hemoglobin (g/L) | 0.996 (0.986,1.008) | 0.526 |
| Hematocrit % | 0.983 (0.947,1.020) | 0.356 |
| Hypertension: Yes vs No | 0.700 (0.309,1.582) | 0.391 |
| Diabetes: Yes vs No | 0.967 (0.520,1.798) | 0.915 |
| Shock: Yes vs No | 8.319 (4.329,15.985) | < 0.001 |
| Sepsis: Yes vs No | 3.944 (2.078,7.488) | < 0.001 |
| Time.to.debridement: >12 h vs ≤12 h | 0.399 (0.203,0.785) | 0.0077 |
| LRINEC | 1.209 (1.070,1.366) | 0.002 |
| APACHE II | 1.014 (0.968,1.062) | 0.564 |
| SOFA | 1.061 (0.982,1.146) | 0.132 |
| DBLR | 1.005 (1.002,1.008) | < 0.001 |
| log10(DBLR) | 3.017 (2.007,4.538) | < 0.001 |
| Q1(DBLR<1.936) |  |  |
| Q2 (1.936≤DBLR<5.000) | 2.110 (0.528,8.438) | 0.291 |
| Q3 (5.000≤DBLR<11.615) | 4.567 (1.301,16.029) | 0.018 |
| Q4 (DBLR≥11.615) | 7.859 (2.313,26.696) | < 0.001 |

Note: DBLR, Direct Bilirubin-to-Lymphocyte Ratio; HR, hazard ratio; CI, confidence interval; LRINEC, Laboratory Risk Indicator for Necrotizing Fasciitis; SOFA, Sequential Organ Failure Assessment;APACHE II, Acute Physiology and Chronic Health Evaluation II; ICU, Intensive Care Unit.
