# Supplementary figures and images for: Association between the direct bilirubin to lymphocyte ratio and mortality in patients with necrotizing fasciitis: a retrospective cohort study
Source: Front Med (Lausanne). 2026 Jun 1;13:1841323. doi: 10.3389/fmed.2026.1841323 (PMC13267103; doi:10.3389/fmed.2026.1841323)

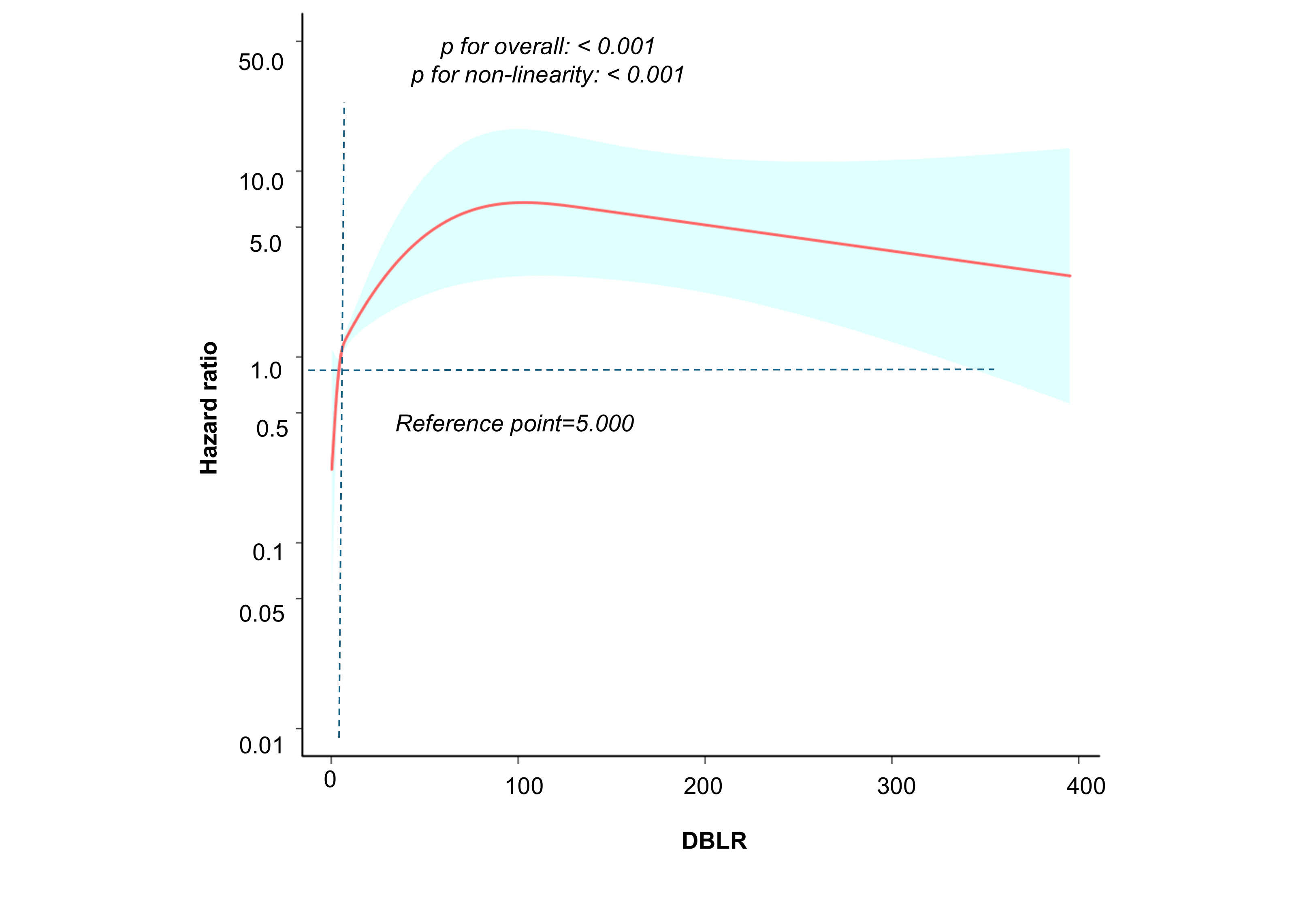

Supplement: Supplementary file 2 [file Image_1.tif]

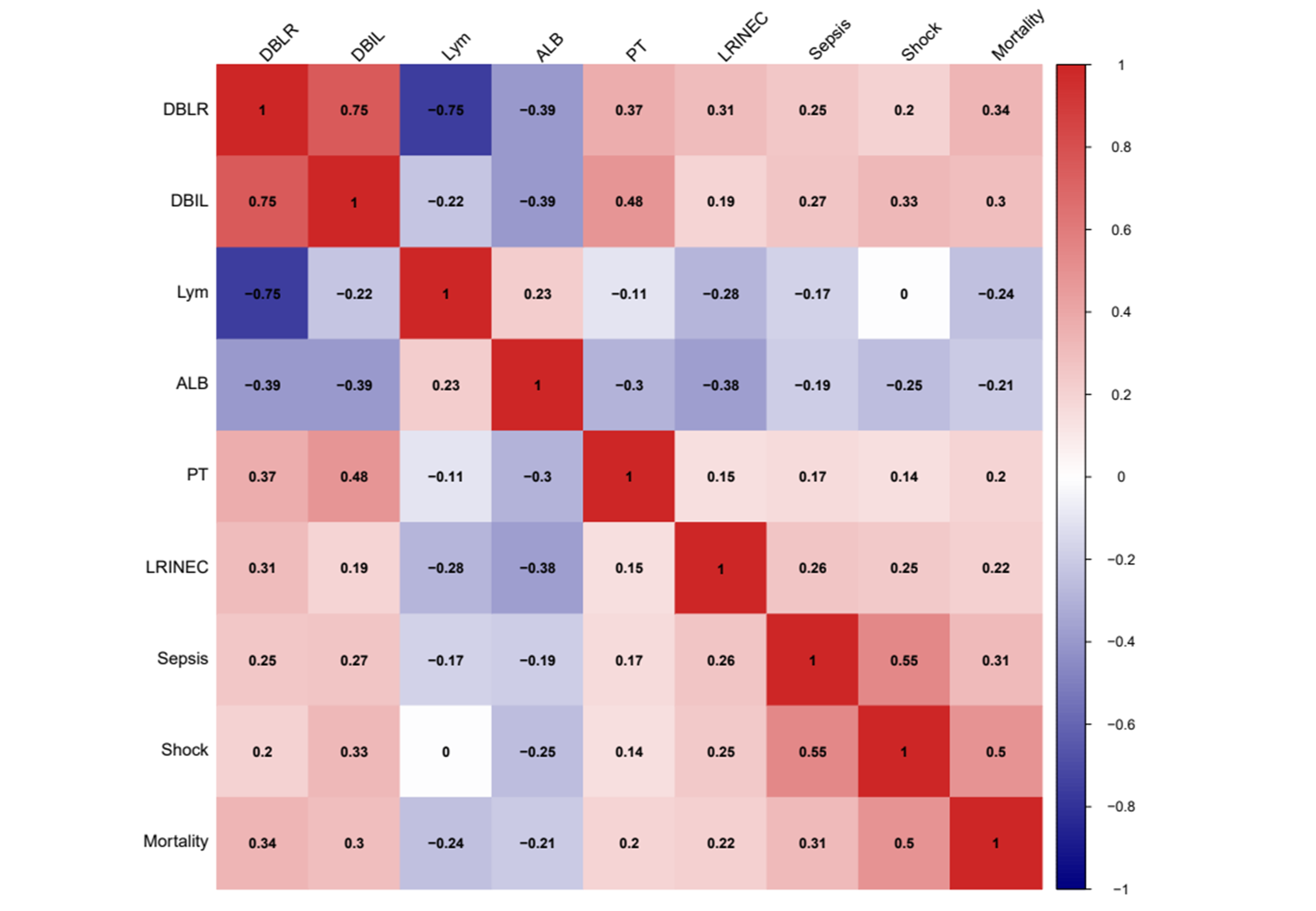

Supplement: Supplementary file 3 [file Image_2.tif]
